# Supplementary figures and images for: Action Observation Training for Upper Limb Stroke Rehabilitation: A Pilot Study on the Role of Attention
Source: J Clin Med. 2025 Sep 19;14(18):6618. doi: 10.3390/jcm14186618 (PMC12471024; doi:10.3390/jcm14186618)

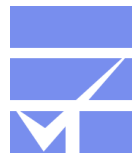

## CONSORT 2010 Flow Diagram

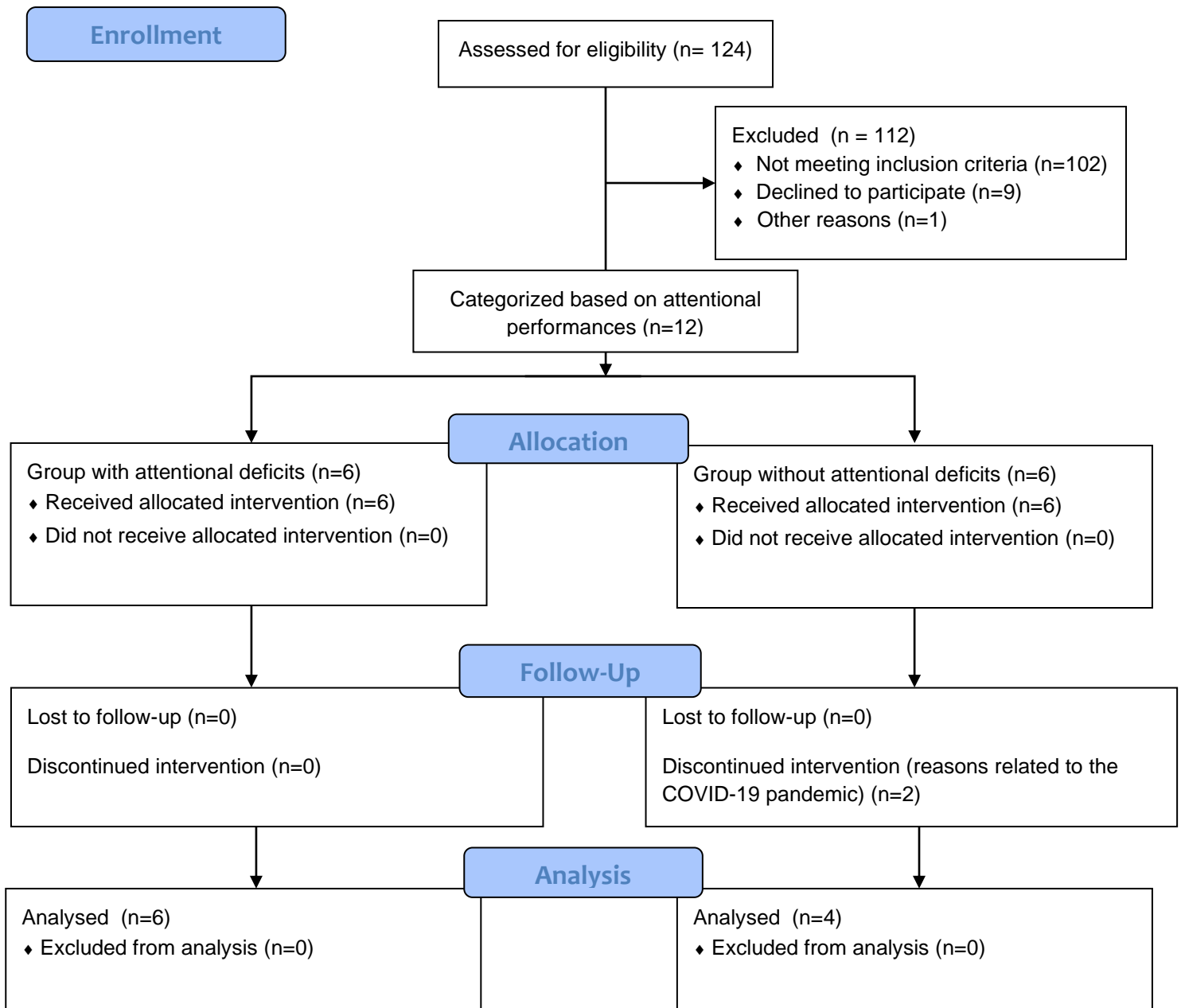

Supplement: Supplementary file 1 [file jcm-14-06618-s001.zip › jcm-3845295 - consort-2010-flow-diagram.pdf]
